# Tracking mood symptoms across the menstrual cycle in women with depression using ecological momentary assessment and heart rate variability

**DOI:** 10.1136/bmjment-2025-301674

**Published:** 2025-06-24

**Authors:** Kyra Delray, Glyn Lewis, Joseph F Hayes

**Affiliations:** 1 University of Oxford Big Data Institute, Oxford, UK; 2 Division of Psychiatry, University College London, London, UK

**Keywords:** Depression & mood disorders

## Abstract

**Background:**

There is limited research on premenstrual exacerbation (PME) of depression. It is unclear how mood and fatigue fluctuate across the menstrual cycle, and whether heart rate variability (HRV) tracks these fluctuations.

**Objective:**

To determine if there is PME of mood, energy and HRV in depressed women.

**Methods:**

Cohort study in women with depression, using the mobile health platform, Juli, to track their menstrual cycle, HRV, mood and energy using ecological momentary assessment (EMA). We modelled the relationship between mood, energy, HRV and menstrual cycle with different lag times (0–3 days) using simple polynomial regression. Results are reported as the SD change from the average rating for an individual for each day across the menstrual cycle.

**Findings:**

Women diagnosed with depression (N=352) tracked their menstrual cycle (≥2 periods), HRV and recorded ≥5 daily mood and energy levels (N=9393 entries). We found a gradual decline in mood beginning at 14 days before menstruation and continuing until 3 days before the next menstruation (β=0.0004, 95% CI 0.0001 to 0.0008, p<0.001). Mood ratings were lowest from 3 days before until 2 days after menstruation; 54.3% (95% CI 48.9% to 59.6%) had a lower mean score during this period than the rest of the cycle. Through the rest of the cycle, participants experienced improvement in mood. Mood rating was associated with HRV on the same day (β=−0.0022, 95% CI −0.0020 to −0.0026, p=0.005) and 1–3 days prior. Energy was not associated with the day of the menstrual cycle.

**Conclusions:**

There is variation in mood across the menstrual cycle in women with depression, consistent with PME.

**Clinical implications:**

EMA over two consecutive cycles could be useful for understanding menstrual cycle-related mood changes and diagnostic clarity may lead to alternative treatment and management options.

**WHAT IS ALREADY KNOWN ON THIS TOPIC**

- A recent review of premenstrual exacerbation of depression found inconsistent findings, with several high-quality studies suggesting no relationship between depression symptoms and menstrual cycle or hormone levels.

**WHAT THIS STUDY ADDS**

- In this cohort study of 352 women, we observed a gradual decline in daily mood ratings, beginning at 14 days before menstruation and continuing until 3 days before the next menstruation. During the remainder of the cycle, participants experienced a gradual improvement in mood. Mood rating was associated with heart rate variability on the same day and 3 days before. Energy was not associated with day of menstrual cycle.

**HOW THIS STUDY MIGHT AFFECT RESEARCH, PRACTICE OR POLICY**

- Premenstrual exacerbation of depression is present in women with depression, similar to the pattern seen in premenstrual dysphoric disorder. Clinically, ecological momentary assessment of mood over at least two consecutive cycles could be a useful tool for understanding menstrual cycle-related mood changes and diagnostic clarity may lead to alternative treatment and management options.

## Introduction

 Premenstrual disorders (PMDs) include premenstrual syndrome (PMS) and premenstrual dysphoric disorder (PMDD). PMS is defined in the ICD-11 as the presence of negative symptoms, including low mood, in the luteal phase.^1^ Some studies have found that this affects 47% of the world’s female population.^2^ PMDD is a more severe and impairing condition, classified as five or more symptoms including low mood and other depressive symptoms in the week before the onset of menses that interfere with daily function. This is a disorder that affects 1.6% of the population.^3^ It is believed that PMS and PMDD are caused by ovarian hormonal fluctuations. Previous research has reported daily expected levels of these hormones; however, the precise aetiology remains unclear.^1^

PMDs are differentiated from major depressive disorders in diagnostic manuals, with PMD symptoms specifically tied to phases of the menstrual cycle and depression present throughout the cycle. Therefore, premenstrual exacerbations (PME) in mood in women with depression are under-researched.^4^ There are no clear guidelines to differentiate PMD from PME. A recent review found inconsistent findings, with several high-quality studies suggesting no relationship between depression symptoms and menstrual cycle or hormone levels.^5^ Other studies suggest mood symptoms worsening in women with depression in the premenstrual phase, with a prevalence of up to 80%.^6^ However, the nature, timing and mechanisms of changes are not well characterised.^7^

Heart rate variability (HRV) is a measure of the variation in time between each heartbeat. It is a measure of autonomic activity that has been linked to a variety of mental health problems, including depression.^8^ Previous research has found an association between HRV and mood where depressed mood is related to a lower HRV and HRV varies with depression severity.^9 10^ There is also an association between HRV and energy or fatigue.^11^ Studies have found that HRV changes in accordance with the menstrual cycle, in line with sympathetic and parasympathetic changes across the cycle.^12^,^14^ HRV decreases before menses and increases afterwards, with changes more prominent in women with PMDs.^14^

We hypothesised that we would observe PME in women with depression: that mood and energy would be lower in the week prior to menses, in keeping with findings in PMDs and that HRV would also be reduced during this phase.

## Methods

### Data source

Advances in smartphones, wearables and mobile health applications permit the regular capture of mood data in real-world situations (ecological momentary assessment; EMA) and the potential to pair this with passively collected data (such as movement, sleep and HRV). Juli is a mobile health platform that helps users with depression monitor their symptoms, understand exacerbating factors and receive behavioural interventions. Juli is available on iOS and Android worldwide. Users consent to their aggregated deidentified data being used for research during the onboarding process.

### Participants

Participants were women over the age of 18, using the Juli platform, who stated that they had a clinical diagnosis of depression. Depression was confirmed using the Patient Health Questionnaire (PHQ-8), with participants scoring >4 at baseline.^15^ Participants needed to have recorded at least two menstrual cycles and made at least five mood/energy ratings to be included in the cohort, so we could examine intraparticipant variation.

### Mood and energy measures

**Figure 1:**
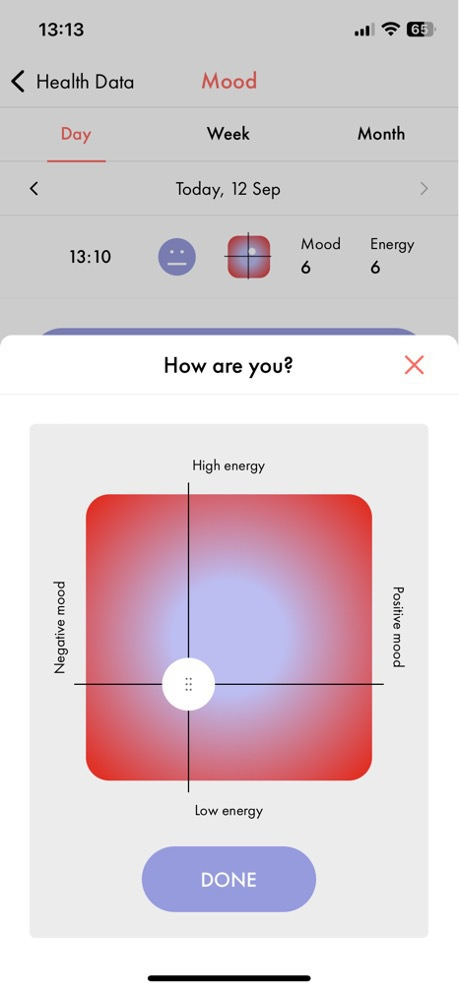
Circumplex model of mood and energy.

Participants were invited to record their mood and energy levels at least once per day via push notifications, participants making five or more entries on different days were included in the study. They recorded their mood and energy levels using a modified circumplex model,^16^ with mood on the X-axis and energy on the Y-axis via a touchscreen interface (figure 1). For the purposes of this study, mood and energy were analysed separately, with values ranging from 1 to 7, lower values represented worse mood or lower energy.

Every 2 weeks, the participants completed the PHQ-8 via the smartphone platform, where they were asked about depression symptoms in the preceding 2 weeks. The PHQ is a widely used, self-completed, depression scale that corresponds to the DSM-IV criteria for a major depressive episode and is more sensitive to change than other depression questionnaires, such as the Hamilton Rating Scale for Depression or the Beck Depression Inventory.^17^ The PHQ-8 excludes the PHQ-9 question about suicidality, and it is therefore preferred in studies where there is no face-to-face patient contact. Deletion of this question has little effect on the scale’s psychometric properties because this question is the least frequently endorsed item on the PHQ-9. Subsequently, the PHQ-8 and PHQ-9 have identical scoring thresholds for depression severity, with higher scores representing more severe depression.^15 18^ The sensitivity and specificity of a PHQ-8 score ≥10 for major depressive disorder is 100% and 95%, respectively.^15^

### Heart rate variability

HRV data were captured from participants via smartphone-based camera applications or smart devices. HRV is reported as SD of the inter-beat intervals (SDNN) measured in milliseconds (ms). We used SDNN, rather than other measures of HRV, as it reflects both short-term and long-term HRV and is influenced by parasympathetic and sympathetic activity. It is also less susceptible to differences by measurement method.^19^ Devices vary in the daily timing of HRV measurement and recording length, we attempted to standardise this as much as possible by asking participants to record HRV on waking, in a sitting position. Where there was more than one HRV measure we used the first of the day. Because all comparisons are within individual, we assumed they used a consistent method of HRV recording throughout the study. SDNN varies by age, sex and a range of health-related characteristics. However, in the healthy population, it ranges from 32 to 93 ms (mean 50, SD 16).^20^

### Menstrual cycle

We created a variable, ‘cycle day’, which spans −14 to +20 days, with day 0 marking the first day of a menstrual period. Women’s menstrual cycles vary in length, however the luteal phase, postovulation, is consistently 14 days long; therefore, we retrospectively used this to align the days of the cycle of the participants for modelling. The negative valued days relate to the luteal phase and the positive to the follicular phase. Cycle lengths outside the usual normal range of 21–35 days were excluded, so the end of observations, per user will vary from +7 to +20. We can assume therefore the total number of women in the study, and therefore the number of mood entries per day, will decrease from day 7 upwards.

### Statistical analysis

We explored the demographic characteristics of the data and used polynomial regression to understand the relationship between menstrual cycle day and number of EMA mood and energy entries on that day (reporting the F-statistic, p value and adjusted R^2^ for this association). To verify the clinical relevance of the daily mood and energy measurement, we used linear regression to quantify the association between the PHQ-8 score and the daily mood/energy score, on the days on which participants completed both (reporting the β-coefficient, 95% CI and p value for the association, which represents the change in mood/energy score for each one point increase in PHQ-8 score).

We modelled cycle day as a predictor of mood and energy using polynomial regression. We examined linear, quadratic, cubic and quartic models. The null hypothesis was that mood was constant across the menstrual cycle and varying HRV levels. We normalised the outcome variables: mood and energy, for everyone, so we were predicting the change from the participants’ mean in SD from their mean. These models allow for flexibility while maintaining simplicity and interpretability, with participants only being compared with themselves. We also investigated models with confounders using multiple polynomial regression. Results are reported as the SD change from the average mood score for an individual for each day across the menstrual cycle (reporting the β-coefficient, 95% CI and p value for the association).

We used the same modelling approach to examine the relationship between HRV and mood/energy. We considered lag periods of 0, 1, 2, 3 days for the association between HRV and mood/energy.

All analyses were completed using R.

## Results

In total, 352 women with depression tracked their menstrual cycle across two cycles, had HRV values and recorded daily mood and energy with the app for at least 5 days. These women made 9346 mood/energy entries. An additional 241 women with depression were excluded as they made fewer than five mood/energy entries. The median age of women included in the study was 28 (IQR 23–33), 141 (40.1%) were white, 170 (48.3%) had ethnicity data missing. 253 (71.9%) were prescribed an antidepressant at baseline and 196 (55.7%) had received a depression diagnosis more than 5 years before entry to the study. The mean mood and energy score across all entries were 4.3 (SD 1.51) and 3.62 (SD 1.50) out of 7, respectively. Mean HRV was 40.7 ms (SD 17.8 ms). Mean PHQ-8 score was 11.30 (SD 6.24). Daily mood responses were associated with total PHQ-8 scores (β=−0.037, 95% CI −0.046 to −0.028, p=0.004), as were daily energy responses (β=−0.92, 95% CI −1.04 to −0.79, p<0.001).

**Figure 2:**
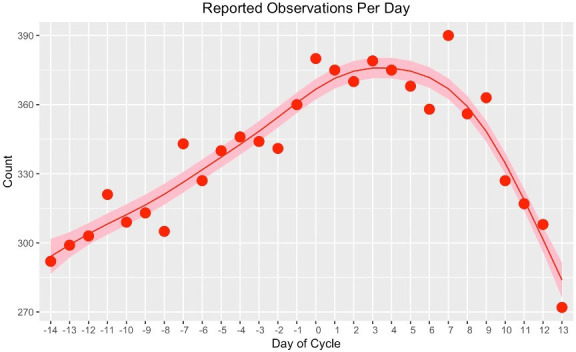
General additive modelling of number of daily EMA mood and energy entries by day of cycle in the total study population. EMA, ecological momentary assessment.

There was strong statistical evidence of a relationship between the number of EMA mood and energy entries and the cycle day, with number of entries peaking on day 3 (F=244.1, p<0.001, adjusted R^2^=0.98) (figure 2). In this model, the day of the cycle explained 87% of the variance in the rate of response.

**Figure 3:**
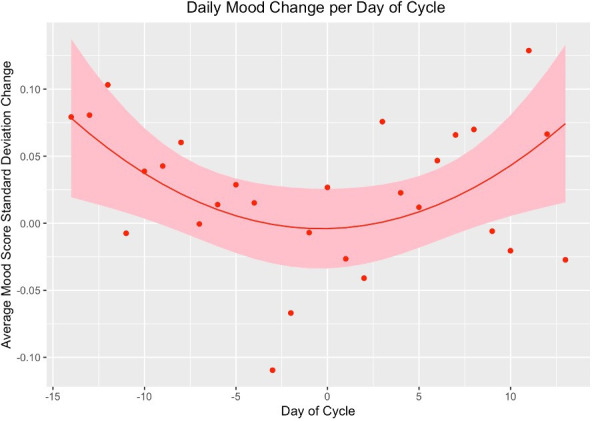
Polynomial linear regression, mood and day of cycle.

Using polynomial regression, we found a consistent decline in mood from 14 days before menstruation until approximately 3 days before the start of the next menstrual cycle (β=0.0004, 95% CI 0.0001 to 0.0008, p<0.001). Daily mood change, in SD from the participants’ mean, for each day of the cycle is shown in figure 3. Mood was worse than the participants’ average from day 3 to day 2 of the cycle (mean difference 0.13, 95% CI 0.02 to 0.25, p=0.022). Of the 352 participants, 191 (54.3%, 95% CI 48.9% to 59.6%) had a lower mean score during this period than the rest of the cycle. Mood score and HRV were significantly linearly related (β=−0.0022, 95% CI −0.0020 to −0.0026, p=0.005) on the same day, with a similar association with a lag of 1–3 days (p=0.005). We found no relationship between energy and cycle day (β=0.0002, 95% CI −0.0006 to 0.0004, p=0.7).

## Discussion

As far as we are aware, this is the first study to characterise daily mood changes over the course of the menstrual cycle using EMA in women with diagnosed depression. We found that mood was associated with the day of the menstrual cycle; this involved a gradual non-linear decline from 14 days before the next cycle to 3 days prior to the start of the next cycle. Mood gradually improved from 2 days after the start of the cycle. Over half of women had a worse mood in the period from 3 days before until 2 days after menstruation, than their average mood across the rest of the cycle.

This suggests that PME is present in women with major depression, similar to the pattern seen in PMD. We found no association between energy level and cycle day. HRV was associated with mood on the same day and 3 days into the future, suggesting HRV may be a proxy biomarker for mood fluctuations in depressed women. Rate of EMA completion was also highly correlated with menstrual cycle day. This was unexpected and should be considered in future EMA studies.

### Strengths and limitations

This study’s main strength was its ability to collect EMA data on large number of participants with depression daily, which allowed us to observe nuanced daily variation and trends across the menstrual cycle. The mood and energy scales are not widely used in clinical settings though they are very similar to visual analogue scales that are commonly used and we found them to be highly correlated with PHQ-8 score.

The analysis is within person, so confounding is limited. Any possible confounding based on the user was adjusted for using random effect modelling and it is quite impossible that reverse causality exists. Only people with access to smartphones and the ability to see a doctor to be diagnosed with depression were included; therefore, participants were likely to be of a higher socioeconomic background than the general population. However, it is unlikely that socioeconomic background or access to smartphones affects the length and effects of the menstrual cycle, so these are not confounders. We were lacking important baseline information about the participants; including their additional health problems and medication, sociodemographic details and detailed clinical assessment of their depression because the study was ‘all remote’ and the data were collected in the context of self-monitoring of symptoms. Ethnicity data were missing for a large minority of the participants. However, these will not confound the relationship between menstrual cycle and daily mood within an individual. Unfortunately, we did not have information about whether participants were prescribed hormone contraception. Hormone contraception can improve PMD symptoms, but studies are conflicting on their impact on depression.^21^ Previous studies have found similar rates of PME in depressed women taking hormone contraception and those not prescribed contraception.^22^ We did not verify ovulation in the study participants, this is estimated from the date of menstruation, with a luteal phase of 14 days.^23^ Future studies to determine the relationship between hormonal changes and PME should confirm ovulation via blood tests.

The measure of HRV would have varied between participants but should have varied little within data from the same participant. This makes comparison with previous literature challenging, however, the mean SDNN we found is consistent with the existing studies of depressed individuals.^24^

### Implications

The PME of mood we observed follows a similar pattern to that seen in women with PMDs without depression and coincides with the rapid fall in oestrogen and progesterone that occurs in the late luteal phase. The precise aetiology of PMDs remains unclear. It has been suggested that PMD symptoms are driven by a pathological sensitivity to withdrawal of allopregnanolone, a progesterone metabolite and anxiolytic neurosteroid, and/or that they are due to a deficit in the functioning of the serotonin transporter.^25^ Importantly, serotonin reuptake inhibitors (SSRIs), which are effective treatments for PMDs, may work through either of these mechanisms.^25^ Unlike the SSRI response in major depression, the response in PMDs occurs within days, suggesting they may work through a different mechanism.^26^ Potentially due to increased sensitivity to synaptic serotonin in PMD or the increased production of allopregnanolone.^26^ The majority of women included in our study were prescribed an SSRI but still displayed PME. These women may be particularly vulnerable to menstrual cycle-related serum level variability of drugs and may therefore need specific drug monitoring and adaptation.^27^ There is a limited evidence-base for reating PME with variable dosing of SSRIs and studies of augmentation with hormonal contraceptives have had conflicting results^28^

We did not find an association between energy and menstrual cycle day. However, previous studies have found that fatigue is a common symptom in PMDs.^29^ This may suggest that women with depression have less fluctuation in energy across the menstrual cycle and that fatigue is not a unique symptom of PME. One previous study compared depressed women self-reporting PME to depressed women without PME and found similar rates of energy/fatigue symptoms (92% vs 93%).^22^

HRV has previously been found to be associated with PMD, with decreased HRV observed in the luteal phase only in women with PMD.^13^ We found similar changes in our study, with HRV tracking mood. HRV biofeedback may be a suitable intervention for PME, with positive impacts on depression and anxiety symptoms.^30^

There remains a lack of diagnostic certainty in how to differentiate PMDs and PME.^7^ However, PME predicts non-response to treatment, a more chronic or recurrent course of illness and impaired functioning in depression.^21^ As such, EMA of mood over at least two consecutive cycles could be a useful clinical tool for understanding menstrual cycle-related mood changes and diagnostic clarity may lead to alternative treatment and management options. Research into the epidemiology, mechanisms and treatment for PME of depression is currently insufficient. Given the huge burden for affected women, there is a clear need for a comprehensive assessment of menstrual cycle-related changes in women with depression.

## Data Availability

Data are available upon reasonable request.
